# Supplementary material for: Complex Multimorbidity and Incidence of Long-Term Care Needs in Japan: A Prospective Cohort Study
Source: Int J Environ Res Public Health. 2021 Oct 7;18(19):10523. doi: 10.3390/ijerph181910523 (PMC8508235; doi:10.3390/ijerph181910523)
Supplement: Supplementary file 1 [file ijerph-18-10523-s001.zip › ijerph-1317490-supplementary.pdf]

Table S1. 44 variables used in the calculation of the propensity score.

**Age**	
Sex	
Have you ever had a check-up at a health center, your workplace, a medical institution, or another place?	1. I had one within a year<br>2. I had one within 2 to 3 years<br>3. I had one more than 4 years ago<br>4. I've never had one
What is the status of your dental health?	1. I have 20 or more natural teeth<br>2. I have 10 to 19 natural teeth<br>3. I have 1 to 9 natural teeth<br>4. I have no natural teeth
How often did you eat meat or fish over the past month?	1. Twice a day or more<br>2. Once a day<br>3. Four to six times a week<br>4. Two or three times a week<br>5. Once a week<br>6. Less than once a week<br>7. None
How often do you eat fruits and vegetables over the past month?	1. Twice a day or more<br>2. Once a day<br>3. Four to six times a week<br>4. Two or three times a week<br>5. Once a week<br>6. Less than once a week<br>7. None
How many years of formal education have you had?	1. Less than 6 years<br>2. 6 to 9 years<br>3. 10 to 12 years<br>4. 13 years or more<br>5. Other
What is your marital status?	1. Married<br>2. Widowed<br>3. Divorced<br>4. Never married<br>5. Other
Who do you live with?	1. I live alone<br>2. I live with someone
What type of residence do you live in?	1. Owned house<br>2. Privately rented house<br>3. Municipally managed house<br>4. Company-owned house<br>5. Other
What is the architectural type of your home?	1. Detached house<br>2. Row house<br>3. Apartment building<br>4. Other
How worried are you if you have to deal with an unexpected expense?	1. Not at all<br>2. Slightly<br>3. Moderately<br>4. Very
Are you receiving a pension?	1. Yes<br>2. No
What is your current working status?	1. I have a paid job.<br>2. I am retired from my job.<br>3. I have never had a job
Who do you usually have meals with?	1. I eat meals alone.<br>2. I eat meals with someone.
Do you currently drink alcohol?	1. Yes<br>2. I used to drink<br>3. No
Do you smoke cigarettes?	1. I have never smoked<br>2. I stopped smoking 5 or more years ago<br>3. I stopped smoking within the past 4 years

	4. I am currently a smoker
Have you had any falls over the past year?	1. Many times<br>2. Once<br>3. None
Are you very worried about falls?	1. Yes<br>2. No
Do you go up stairs without holding onto the handrail or the wall?	1. Yes<br>2. No
Do you get up out of chairs without holding anything?	1. Yes<br>2. No
How long do you walk a day on average?	1. Less than 30 minutes<br>2. 30 to 59 minutes<br>3. 60 to 89 minutes<br>4. 90 minutes or more
How often do you go out?	1. Almost everyday<br>2. Two or three times a week<br>3. Once a week<br>4. Once or twice a month<br>5. Several times a year<br>6. Rarely
Has the frequency of your going out decreased since last year?	1. Yes<br>2. No
Do you engage in any leisure activities at the moment?	1. Yes<br>2. No
Do you think people living in your area can be trusted in general?	1. Very<br>2. Moderately<br>3. Neutral<br>4. Slightly<br>5. Not at all
Do you think people living in your area try to help others in most situations?	1. Very<br>2. Moderately<br>3. Neutral<br>4. Slightly<br>5. Not at all
How attached are you to the area you live?	1. Very<br>2. Moderately<br>3. Neutral<br>4. Slightly<br>5. Not at all
Do you agree with making it a rule to offer half a day for the interests of the whole area but not for your own interests?	1. I agree<br>2. Neutral<br>3. I disagree
How uneasy do you feel about safety in your community?	1. Very uneasy<br>2. More or less uneasy<br>3. Slightly uneasy<br>4. Not uneasy at all
Do you want to participate in local events or festivals positively?	1. Yes<br>2. No
What kind of interactions do you have with people in your neighborhood?	1. Mutual consultation, lending and borrowing daily commodities, cooperation in daily life<br>2. Standing and chatting frequently<br>3. No more than exchanging greetings<br>4. None, not even greetings
- Are the following present within 1 km of your home? Locations with noticeable graffiti or undisposed garbage	1. Many<br>2. Some<br>3. Few<br>4. None<br>5. I don't know
Are the following present within 1 km of your home? Parks or foot paths suitable for exercise or walking	1. Many<br>2. Some<br>3. Few<br>4. None<br>5. I don't know
Are the following present within 1 km of your home? Locations difficult for walking, such as hills or steps	1. Many<br>2. Some

	3. Few<br>4. None<br>5. I don't know
Are the following present within 1 km of your home?<br>Roads or crossroads with a great risk of traffic accidents	1. Many<br>2. Some<br>3. Few<br>4. None<br>5. I don't know
Are the following present within 1 km of your home?<br>Fascinating views or buildings	1. Many<br>2. Some<br>3. Few<br>4. None<br>5. I don't know
Are the following present within 1 km of your home?<br>Shops or facilities selling fresh fruits and vegetables	1. Many<br>2. Some<br>3. Few<br>4. None<br>5. I don't know
Are the following present within 1 km of your home?<br>Dangerous places when walking alone at night	1. Many<br>2. Some<br>3. Few<br>4. None<br>5. I don't know
Are the following present within 1 km of your home?<br>Houses or facilities you feel free to drop in	1. Many<br>2. Some<br>3. Few<br>4. None<br>5. I don't know
Do you have someone who listens to your concerns and complaints?	1. Yes<br>2. No
Do you have someone who looks after you when you are sick and confined to a bed for a few days?	1. Yes<br>2. No
How often do you attend activities for the following groups?<br>Sports group or club	1. Almost everyday<br>2. Two or three times a week<br>3. Once a week<br>4. Once or twice a month<br>5. A few times a year<br>6. Never
How often do you attend activities for the following groups?<br>Leisure activity group	1. Almost everyday<br>2. Two or three times a week<br>3. Once a week<br>4. Once or twice a month<br>5. A few times a year<br>6. Never

Table S2. Basement Characteristics of populations with or without multimorbidity (MM).

	**With MM (*N* = 20233)**				**Without MM (*N* = 18656)**			
**Characteristic**	Care need		No care need		Care need		No care need	
**Sample size. No**	5588		14645		3580		15076	
Age								
65–69	377	(7)	3710	(25)	313	(9)	5015	(33)
70–74	910	(16)	4763	(33)	641	(18)	5104	(34)
75–79	1571	(28)	3842	(26)	1013	(28)	3121	(21)
80–84	1660	(30)	1758	(12)	961	(27)	1391	(9)
85–89	837	(15)	485	(3)	495	(14)	375	(2)
90–	233	(4)	87	(1)	157	(4)	70	0
missing	0	0	0	0	0	0	0	0
Sex								
male	2234	(40)	6569	(45)	1662	(46)	7376	(49)
female	3354	(60)	8076	(55)	1918	(54)	7700	(51)
missing	0	0)	0	0	0	0	0	0
Previous Health check-up								
I had one within a year	2838	(51)	8888	(61)	1696	(47)	9204	(61)
Ihadonewithin2to3years	672	(12)	1757	(12)	424	(12)	1808	(12)
Ihadonemorethan4yearsago	632	(11)	1426	(10)	379	(11)	1527	(10)
I've never had one	1054	(19)	1962	(13)	815	(23)	2053	(14)
Missing	392	(7)	612	(4)	266	(7)	484	(3)
The number of natural teeth								
20 or more	1127	(20)	4852	(33)	787	(22)	5526	(37)
10 to 19	1153	(21)	3793	(26)	756	(21)	3930	(26)
1 to 9	1758	(31)	3720	(25)	1071	(30)	3511	(23)
Non-natural teeth	1312	(23)	1862	(13)	796	(22)	1688	(11)
missing	238	(4)	418	(3)	170	(5)	421	(3)
Consumption of meat and fish								
Twice a day or more	411	(7)	1114	(8)	264	(7)	1130	(7)
Once a day	1652	(30)	4465	(30)	1090	(30)	4636	(31)
Four six times a week	1147	(21)	3347	(23)	733	(20)	3382	(22)
Two or three times a week	1517	(27)	3856	(26)	916	(26)	3963	(26)
Once a week	305	(5)	752	(5)	215	(6)	763	(5)
Less than once a week	136	(2)	248	(2)	77	(2)	266	(2)
None	38	(1)	42	0	13	0	35	0
missing	382	(7)	821	(6)	272	(8)	901	(6)
Consumption of fruits and vegetables								
Twice a day or more	2366	(42)	6641	(45)	1464	(41)	6796	(45)
Once a day	1762	(32)	4604	(31)	1156	(32)	4651	(31)
Four six times a week	618	(11)	1583	(11)	415	(12)	1757	(12)
Two or three times a week	386	(7)	878	(6)	275	(8)	883	(6)
Once a week	63	(1)	122	(1)	32	(1)	115	(1)
Less than once a week	46	(1)	46	0	16	0	52	0
None	10	0	21	0	5	0	11	0
missing	337	(6)	750	(5)	217	(6)	811	(5)
Formal educational year								
Lessthan6years	297	(5)	285	(2)	200	(6)	212	(1)
6to9years	2821	(50)	6991	(48)	1688	(47)	6609	(44)
10to12years	1527	(27)	4707	(32)	1028	(29)	5222	(35)
13yearsormore	719	(13)	2372	(16)	492	(14)	2716	(18)
Other	49	(1)	76	(1)	29	(1)	93	(1)
missing	175	(3)	214	(1)	143	(4)	224	(1)
Marital status								
Married	3182	(57)	10,373	(71)	2137	(60)	11,191	(74)
Widowed	1948	(35)	3176	(22)	1125	(31)	2819	(19)
Divorced	178	(3)	474	(3)	106	(3)	488	(3)

Never married	118	(2)	295	(2)	67	(2)	264	(2)
Other	23	0	85	(1)	22	(1)	85	(1)
missing	139	(2)	242	(2)	123	(3)	229	(2)
Someone living with								
live alone	4515	(81)	12,680	(87)	2956	(83)	13,213	(88)
not alone	944	(17)	1786	(12)	507	(14)	1631	(11)
missing	129	(2)	179	(1)	117	(3)	232	(2)
Residence type								
Owned house	4722	(85)	12,740	(87)	3039	(85)	13,172	(87)
Privately rented house	240	(4)	597	(4)	177	(5)	619	(4)
Municipally managed house	309	(6)	748	(5)	166	(5)	744	(5)
Company-owned house	5	0	25	0	3	0	24	0
Other	77	(1)	176	(1)	32	(1)	159	(1)
missing	235	(4)	359	(2)	163	(5)	358	(2)
Architectural type of home								
Detached house	4490	(80)	12,200	(83)	2932	(82)	12,586	(83)
Rowhouse	126	(2)	312	(2)	90	(3)	282	(2)
Apartment building	528	(9)	1416	(10)	316	(9)	1507	(10)
Other	60	(1)	131	(1)	25	(1)	123	(1)
missing	384	(7)	586	(4)	217	(6)	578	(4)
Worries about unexpected expenses								
Not at all	584	(10)	1274	(9)	480	(13)	1538	(10)
Slightly	2190	(39)	6028	(41)	1467	(41)	6890	(46)
Moderately	1443	(26)	4113	(28)	835	(23)	3866	(26)
Very	974	(17)	2457	(17)	461	(13)	2033	(13)
missing	397	(7)	773	(5)	337	(9)	749	(5)
Receiving pension								
no	5237	(94)	13,954	(95)	3361	(94)	14,418	(96)
yes	84	(2)	193	(1)	44	(1)	165	(1)
missing	267	(5)	498	(3)	175	(5)	493	(3)
Current working status								
having a paid job	497	(9)	2762	(19)	398	(11)	3657	(24)
retired	3060	(55)	8284	(57)	1941	(54)	8099	(54)
never having a job	956	(17)	1667	(11)	525	(15)	1518	(10)
missing	1075	(19)	1932	(13)	716	(20)	1802	(12)
Persons to have meal with								
No one	3676	(66)	10,781	(74)	2492	(70)	11,461	(76)
With someone	1569	(28)	3136	(21)	859	(24)	2781	(18)
missing	343	(6)	728	(5)	229	(6)	834	(6)
Alcohol								
Yes	1173	(21)	4467	(31)	893	(25)	5271	(35)
Used to drink	259	(5)	581	(4)	163	(5)	465	(3)
No	3728	(67)	8770	(60)	2262	(63)	8471	(56)
missing	428	(8)	827	(6)	262	(7)	869	(6)
Smoking								
I have never smoked	3042	(54)	7948	(54)	1824	(51)	8018	(53)
Istoppedsmoking5ormoreyears ago	1158	(21)	3341	(23)	727	(20)	3359	(22)
Istoppedsmokingwithinthepast 4years	214	(4)	699	(5)	163	(5)	736	(5)
I am currently a smoker	452	(8)	1180	(8)	390	(11)	1528	(10)
missing	722	(13)	1477	(10)	476	(13)	1435	(10)
Falling over								
Many times	866	(15)	1224	(8)	397	(11)	708	(5)
Once	1609	(29)	3676	(25)	913	(26)	3085	(20)
None	2806	(50)	9119	(62)	2078	(58)	10,673	(71)
missing	307	(5)	626	(4)	192	(5)	610	(4)

Worries about falls								
Yes	3626	(65)	7053	(48)	1882	(53)	5579	(37)
No	1556	(28)	6721	(46)	1373	(38)	8569	(57)
missing	406	(7)	871	(6)	325	(9)	928	(6)
Going upstairs without support								
Yes	1953	(35)	7801	(53)	1586	(44)	9740	(65)
No	3332	(60)	6217	(42)	1808	(51)	4706	(31)
missing	303	(5)	627	(4)	186	(5)	630	(4)
Get up out of chairs without support								
Yes	3192	(57)	11,492	(78)	2449	(68)	12,902	(86)
No	2098	(38)	2553	(17)	949	(27)	1598	(11)
missing	298	(5)	600	(4)	182	(5)	576	(4)
Average time to walk								
Lessthan30minutes	2553	(46)	4877	(33)	1496	(42)	4256	(28)
30to59minutes	1570	(28)	4894	(33)	1041	(29)	5020	(33)
60to89minutes	565	(10)	2085	(14)	400	(11)	2386	(16)
90minutesormore	402	(7)	1879	(13)	328	(9)	2539	(17)
missing	498	(9)	910	(6)	315	(9)	875	(6)
Frequency of going out								
Almost every day	1885	(34)	7301	(50)	1417	(40)	8424	(56)
Two or three times a week	1779	(32)	4261	(29)	1067	(30)	3910	(26)
Once a week	719	(13)	1349	(9)	393	(11)	1177	(8)
Once or twice a month	525	(9)	718	(5)	269	(8)	519	(3)
Several times a year	112	(2)	133	(1)	76	(2)	128	(1)
Rarely	182	(3)	129	(1)	94	(3)	98	(1)
missing	386	(7)	754	(5)	264	(7)	820	(5)
Decrease in the frequency of going out								
Yes	2459	(44)	3637	(25)	1222	(34)	2516	(17)
No	2696	(48)	10,145	(69)	2083	(58)	11,601	(77)
missing	433	(8)	863	(6)	275	(8)	959	(6)
Engagement in leisure activities								
Yes	2197	(39)	7959	(54)	1483	(41)	8776	(58)
No	2892	(52)	5587	(38)	1766	(49)	5177	(34)
missing	499	(9)	1099	(8)	331	(9)	1123	(7)
Trust in neighbors								
Very	752	(13)	1834	(13)	565	(16)	2089	(14)
Moderately	2804	(50)	7913	(54)	1827	(51)	8415	(56)
Neutral	1358	(24)	3467	(24)	800	(22)	3316	(22)
Slightly	222	(4)	537	(4)	120	(3)	423	(3)
Not at all	57	(1)	130	(1)	30	(1)	97	(1)
missing	395	(7)	764	(5)	238	(7)	736	(5)
Support from neighbors								
Very	438	(8)	968	(7)	332	(9)	1043	(7)
Moderately	2371	(42)	6586	(45)	1574	(44)	7305	(48)
Neutral	1796	(32)	4867	(33)	1074	(30)	4731	(31)
Slightly	408	(7)	1120	(8)	248	(7)	1006	(7)
Not at all	110	(2)	244	(2)	66	(2)	164	(1)
missing	465	(8)	860	(6)	286	(8)	827	(5)
Attachment to residence								
Very	1732	(31)	4355	(30)	1162	(32)	4553	(30)
Moderately	2492	(45)	7195	(49)	1664	(46)	7550	(50)
Neutral	786	(14)	1877	(13)	448	(13)	1914	(13)
Slightly	258	(5)	629	(4)	109	(3)	523	(3)
Not at all	53	(1)	119	(1)	29	(1)	73	0

missing	267	(5)	470	(3)	168	(5)	463	(3)
Contribution to residence								
I agree	1864	(33)	6070	(41)	1254	(35)	6875	(46)
Neutral	2927	(52)	7188	(49)	1790	(50)	6839	(45)
I disagree	303	(5)	568	(4)	171	(5)	500	(3)
missing	494	(9)	819	(6)	365	(10)	862	(6)
Uneasiness about safety in residence								
Very uneasy	410	(7)	1125	(8)	224	(6)	988	(7)
More or less uneasy	2471	(44)	7095	(48)	1443	(40)	6942	(46)
Slightly uneasy	1940	(35)	5141	(35)	1341	(37)	5747	(38)
Not easy at all	350	(6)	611	(4)	267	(7)	704	(5)
missing	417	(7)	673	(5)	305	(9)	695	(5)
Participation in local events								
Yes	1881	(34)	5953	(41)	1260	(35)	6680	(44)
No	3160	(57)	7656	(52)	1882	(53)	7303	(48)
missing	547	(10)	1036	(7)	438	(12)	1138	(8)
Interactions with neighborhood								
Daily Mutual consultation and cooperation	844	(15)	2365	(16)	557	(16)	2499	(17)
Standing and chatting frequently	2831	(51)	8080	(55)	1789	(50)	8425	(56)
No more than exchanging greetings	1250	(22)	2910	(20)	795	(22)	2977	(20)
None not even greetings	111	(2)	179	(1)	88	(2)	156	(1)
missing	552	(10)	1111	(8)	351	(10)	1019	(7)
Near to residence								
Locations with graffiti or garbage								
Many	201	(4)	592	(4)	123	(3)	539	(4)
Some	1209	(22)	3673	(25)	680	(19)	3672	(24)
Few	2442	(44)	7165	(49)	1643	(46)	7644	(51)
None	815	(15)	1750	(12)	546	(15)	1874	(12)
I don't know	590	(11)	938	(6)	352	(10)	816	(5)
missing	331	(6)	527	(4)	236	(7)	531	(4)
Parks or footpaths								
Many	691	(12)	1968	(13)	438	(12)	2218	(15)
Some	2830	(51)	7903	(54)	1812	(51)	8257	(55)
Few	1169	(21)	3219	(22)	757	(21)	3138	(21)
None	350	(6)	791	(5)	208	(6)	737	(5)
I don’t know	292	(5)	365	(2)	169	(5)	325	(2)
missing	256	(5)	399	(3)	196	(5)	401	(3)
Locations difficult for walking								
Many	441	(8)	997	(7)	250	(7)	872	(6)
Some	1872	(34)	4276	(29)	1048	(29)	3933	(26)
Few	2412	(43)	7429	(51)	1633	(46)	8158	(54)
None	410	(7)	1233	(8)	329	(9)	1455	(10)
I don’t know	206	(4)	359	(2)	141	(4)	278	(2)
missing	247	(4)	351	(2)	179	(5)	380	(3)
Risky roads or crossroads for traffic accidents								
Many	666	(12)	1911	(13)	374	(10)	1752	(12)
Some	2960	(53)	7979	(54)	1797	(50)	8048	(53)
Few	1402	(25)	3839	(26)	1014	(28)	4335	(29)
None	77	(1)	180	(1)	67	(2)	213	(1)
I don’t know	250	(4)	356	(2)	146	(4)	340	(2)
missing	233	(4)	380	(3)	182	(5)	388	(3)

Fascinating views or buildings								
Many	312	(6)	856	(6)	169	(5)	870	(6)
Some	1719	(31)	4702	(32)	1103	(31)	4994	(33)
Few	2247	(40)	6416	(44)	1463	(41)	6703	(44)
None	536	(10)	1477	(10)	329	(9)	1418	(9)
I don't know	441	(8)	688	(5)	260	(7)	583	(4)
missing	333	(6)	506	(3)	256	(7)	508	(3)
Shops selling fresh fruits and vegetables								
Many	639	(11)	2145	(15)	468	(13)	2393	(16)
Some	3050	(55)	8478	(58)	1968	(55)	8872	(59)
Few	1134	(20)	2630	(18)	691	(19)	2541	(17)
None	402	(7)	864	(6)	204	(6)	776	(5)
I don't know	135	(2)	157	(1)	82	(2)	124	(1)
missing	228	(4)	371	(3)	167	(5)	370	(2)
Dangerous places walking alone at night								
Many	616	(11)	1615	(11)	310	(9)	1415	(9)
Some	2540	(45)	7371	(50)	1523	(43)	7442	(49)
Few	1371	(25)	3997	(27)	1103	(31)	4656	(31)
None	105	(2)	202	(1)	88	(2)	219	(1)
I don't know	672	(12)	1018	(7)	352	(10)	893	(6)
missing	284	(5)	442	(3)	204	(6)	451	(3)
Comfortable houses or facilities								
Many	88	(2)	282	(2)	78	(2)	324	(2)
Some	1789	(32)	5140	(35)	1219	(34)	5523	(37)
Few	2256	(40)	6322	(43)	1442	(40)	6456	(43)
None	574	(10)	1423	(10)	314	(9)	1284	(9)
I don't know	603	(11)	1046	(7)	336	(9)	1007	(7)
missing	278	(5)	432	(3)	191	(5)	482	(3)
Someone listening to your concerns								
None	4758	(85)	13,067	(89)	3026	(85)	13,504	(90)
Yes	398	(7)	782	(5)	250	(7)	771	(5)
missing	432	(8)	796	(5)	304	(8)	801	(5)
Someone looking after in case of ill								
None	4870	(87)	13,246	(90)	3141	(88)	13,724	(91)
Yes	337	(6)	688	(5)	176	(5)	585	(4)
missing	381	(7)	711	(5)	263	(7)	767	(5)
Attendance								
Sports group or club								
Almost every day	57	(1)	270	(2)	34	(1)	320	(2)
Two or three times a week	227	(4)	1039	(7)	144	(4)	1146	(8)
Once a week	181	(3)	826	(6)	124	(3)	884	(6)
Once or twice a month	108	(2)	539	(4)	88	(2)	681	(5)
A few times a year	94	(2)	514	(4)	94	(3)	611	(4)
Never	3483	(62)	8383	(57)	2108	(59)	8423	(56)
missing	1438	(26)	3074	(21)	988	(28)	3011	(20)
Leisure activity group								
Almost every day	53	(1)	249	(2)	40	(1)	265	(2)
Two or three times a week	260	(5)	1216	(8)	217	(6)	1253	(8)
Once a week	410	(7)	1354	(9)	250	(7)	1575	(10)
Once or twice a month	468	(8)	1741	(12)	303	(8)	1902	(13)
A few times a year	262	(5)	1081	(7)	197	(6)	1263	(8)
Never	2809	(50)	6302	(43)	1691	(47)	6268	(42)
missing	1326	(24)	2702	(18)	882	(25)	2550	(17)

Data are given as No. (%) unless otherwise noted. Multimorbidity(MM)

Table S3. Basement Characteristics of populations with or without complex multimorbidity (CMM).

	With CMM (*N* = 7565)		Without CMM (*N* = 31,324)	
**Characteristic**	**Care need**	No care need	Care need	No care need
Sample size. No	2508	5057	6660	24,664
Age				
65–69	146(6)	1059(21)	544(8)	7666(31)
70–74	376(15)	1579(31)	1175(18)	8288(34)
75–79	714(28)	1448(29)	1870(28)	5515(22)
80–84	758(30)	727(14)	1863(28)	2422(10)
85–89	398(16)	207(4)	934(14)	653(3)
90–	116(5)	37(1)	274(4)	120(0)
missing	0(0)	0(0)	0(0)	0(0)
Sex				
male	937(37)	2114(42)	2959(44)	11,831(48)
female	1571(63)	2943(58)	3701(73)	12,833(52)
missing	0(0)	0(0)	0(0)	0(0)
Previous health check-up				
I had one within a year	1253(50)	3024(60)	3281(65)	15,068(61)
I had one within 2 to 3 years	312(12)	613(12)	784(16)	2952(12)
I had one more than 4 years ago	260(10)	497(10)	751(15)	2456(10)
I've never had one	489(19)	683(14)	1380(27)	3332(14)
missing	194(8)	240(5)	464(9)	856(3)
The number of natural teeth				
20 or more	485(19)	1535(30)	1429(28)	8843(36)
10 to 19	502(20)	1305(26)	1407(28)	6418(26)
1 to 9	818(33)	1352(27)	2011(40)	5879(24)
no natural teeth	592(24)	719(14)	1516(30)	2831(11)
missing	111(4)	146(3)	297(6)	693(3)
Consumption of meat and fish				
Twice a day or more	193(8)	384(8)	482(10)	1860(8)
Once a day	734(29)	1526(30)	2008(40)	7575(31)
Four to six times a week	525(21)	1142(23)	1355(27)	5587(23)
Two or three times a week	656(26)	1343(27)	1777(35)	6476(26)
Once a week	145(6)	261(5)	375(7)	1254(5)
Less than once a week	68(3)	93(2)	145(3)	421(2)
None	15(1)	20(0)	36(1)	57(0)
missing	172(7)	288(6)	482(10)	1434(6)
Consumption of fruits and vegetables				
Twice a day or more	1054(42)	2240(44)	2776(55)	11,197(45)
Once a day	805(32)	1602(32)	2113(42)	7653(31)
Four to six times a week	264(11)	563(11)	769(15)	2777(11)
Two or three times a week	158(6)	306(6)	503(10)	1455(6)
Once a week	35(1)	46(1)	60(1)	191(1)
Less than once a week	31(1)	21(0)	31(1)	77(0)
None	8(0)	11(0)	7(0)	21(0)
missing	153(6)	268(5)	401(8)	1293(5)
Formal educational year				
Less than 6 years	155(6)	141(3)	342(7)	356(1)
6 to 9 years	1301(52)	2517(50)	3208(63)	11,083(45)
10 to 12 years	662(26)	1585(31)	1893(37)	8344(34)
13 years or more	294(12)	703(14)	917(18)	4385(18)
Other	22(1)	30(1)	56(1)	139(1)
missing	153(6)	81(2)	244(5)	357(1)
Marital status				
Married	1356(54)	3416(68)	3963(78)	18,148(74)
Widowed	928(37)	1243(25)	2145(42)	4752(19)
Divorced	92(4)	163(3)	192(4)	799(3)
Never married	57(2)	100(2)	128(3)	459(2)
Other	11(0)	37(1)	34(1)	133(1)
missing	74(3)	98(2)	198(4)	373(2)
Someone living with				
live alone	1992(79)	4308(85)	5479(108)	21,585(88)
not alone	468(19)	691(14)	983(19)	2726(11)
missing	64(3)	58(1)	198(4)	353(1)
Residence type				

Owned house	2104(84)	4336(86)	5657(112)	21,576(87)
Privately rented house	118(5)	208(4)	299(6)	1008(4)
Municipally managed house	149(6)	292(6)	326(6)	1200(5)
Company-owned house	1(0)	8(0)	7(0)	41(0)
Other	38(2)	75(1)	71(1)	260(1)
missing	48(2)	138(3)	300(6)	579(2)
Architectural type of home				
Detached house	1987(79)	4184(83)	5435(107)	20,602(84)
Row house	60(2)	121(2)	156(3)	473(2)
Apartment building	245(10)	502(10)	599(12)	2421(10)
Other	30(1)	43(1)	55(1)	211(1)
missing	98(4)	207(4)	415(8)	957(4)
Worries about unexpected expenses				
Not at all	235(9)	373(7)	829(16)	2439(10)
Slightly	929(37)	1888(37)	2728(54)	11,030(45)
Moderately	666(27)	1478(29)	1612(32)	6501(26)
Very	500(20)	1054(21)	935(18)	3436(14)
missing	186(7)	264(5)	556(11)	1258(5)
Receiving pension				
no	2351(94)	4811(95)	6247(124)	23,561(96)
yes	41(2)	68(1)	87(2)	290(1)
missing	116(5)	178(4)	326(6)	813(3)
Current working status				
having a paid job	190(8)	762(15)	705(14)	5657(23)
retired	1379(55)	2936(58)	3622(72)	13,447(55)
never having a job	478(19)	647(13)	1003(20)	2538(10)
missing	116(5)	712(14)	1330(26)	3022(12)
Persons to have meal with				
No one	1568(63)	3542(70)	4600(91)	18,700(76)
With someone	788(31)	1259(25)	1640(32)	4658(19)
missing	152(6)	256(5)	420(8)	1306(5)
Alcohol				
Yes	479(19)	1389(27)	1587(31)	8349(34)
Used to drink	118(5)	236(5)	304(6)	810(3)
No	1701(68)	3143(62)	4289(85)	14,098(57)
missing	210(8)	289(6)	480(9)	1407(6)
Smoking				
I have never smoked	1400(56)	2795(55)	3466(69)	13,171(53)
I stopped smoking 5 or more years ago	510(20)	1099(22)	1375(27)	5601(23)
I stopped smoking within the past 4 years	89(4)	245(5)	288(6)	1190(5)
I am currently a smoker	174(7)	372(7)	668(13)	2336(9)
missing	335(13)	546(11)	863(17)	2366(10)
Falling over				
Many times	470(19)	567(11)	793(16)	1365(6)
Once	724(29)	1432(28)	1798(36)	5329(22)
None	1180(47)	2813(56)	3704(73)	16,979(69)
missing	134(5)	245(5)	365(7)	991(4)
Worriees about falls				
Yes	1751(70)	2867(57)	3757(74)	9765(40)
No	579(23)	1861(37)	2350(46)	13,429(54)
missing	178(7)	329(7)	553(11)	1470(6)
Going up stairs without support				
Yes	790(31)	2314(46)	2749(54)	15,227(62)
No	1586(63)	2507(50)	3554(70)	8416(34)
missing	132(5)	236(5)	357(7)	1021(4)
Get up out of chairs without support				
Yes	1300(52)	3678(73)	4341(86)	20,716(84)
No	1066(43)	1154(23)	1981(39)	2997(12)
missing	142(6)	225(4)	338(7)	951(4)
Average time to walk				
Less than 30 min	1186(47)	1857(37)	2863(57)	7276(30)
30 to 59 min	675(27)	1629(32)	1936(38)	8285(34)
60 to 89 min	251(10)	653(13)	714(14)	3818(15)
90 min or more	167(7)	565(11)	563(11)	3853(16)

missing	229(9)	353(7)	584(12)	1432(6)
Frequency of going out				
Almost everyday	756(30)	2349(46)	2546(50)	13,376(54)
Two or three times a week	853(34)	1526(30)	1993(39)	6645(27)
Once a week	331(13)	506(10)	781(15)	2020(8)
Once or twice a month	245(10)	297(6)	549(11)	940(4)
Several times a year	53(2)	60(1)	135(3)	201(1)
Rarely	89(4)	53(1)	187(4)	174(1)
missing	181(7)	266(5)	469(9)	1308(5)
Decrease in the frequency of going out				
Yes	1222(49)	1596(32)	2459(49)	4557(18)
No	1095(44)	3162(63)	3684(73)	18,584(75)
missing	191(8)	299(6)	517(10)	1523(6)
Engagement in leisure activities				
Yes	938(37)	2611(52)	2742(54)	14,124(57)
No	1351(54)	2042(40)	3307(65)	8722(35)
missing	219(9)	404(8)	611(12)	1818(7)
Trust in neighbors				
Very	306(12)	597(12)	1011(20)	3326(13)
Moderately	1212(48)	2592(51)	3419(68)	13,736(56)
Neutral	653(26)	1300(26)	1505(30)	5483(22)
Slightly	120(5)	212(4)	222(4)	748(3)
Not at all	33(1)	61(1)	54(1)	166(1)
missing	184(7)	295(6)	449(9)	1205(5)
Support from neighbors				
Very	177(7)	338(7)	593(12)	1673(7)
Moderately	1043(42)	2143(42)	2902(57)	11748(48)
Neutral	823(33)	1714(34)	2047(40)	7884(32)
Slightly	198(8)	436(9)	458(9)	1690(7)
Not at all	58(2)	107(2)	118(2)	301(1)
missing	209(8)	319(6)	542(11)	1368(6)
Attachment to residence				
Very	735(29)	1467(29)	2159(43)	7441(30)
Moderately	1110(44)	2428(48)	3046(60)	12,317(50)
Neutral	381(15)	699(14)	853(17)	3092(13)
Slightly	125(5)	232(5)	242(5)	920(4)
Not at all	32(1)	58(1)	50(1)	134(1)
missing	125(5)	173(3)	310(6)	760(3)
Contribution to residence				
I agree	783(31)	1977(39)	2335(46)	10,968(44)
Neutral	1358(54)	2552(50)	3359(66)	11,475(47)
I disagree	144(6)	220(4)	330(7)	848(3)
missing	223(9)	308(6)	636(13)	1373(6)
Uneasiness about safety in residence				
Very uneasy	218(9)	441(9)	416(8)	1672(7)
More or less uneasy	1122(45)	2520(50)	2792(55)	11,517(47)
Slightly uneasy	824(33)	1649(33)	2457(49)	9239(37)
Not uneasy at all	159(6)	199(4)	458(9)	1116(5)
missing	185(7)	248(5)	537(11)	1120(5)
Participation in local events				
Yes	787(31)	1951(39)	2354(47)	10,682(43)
No	1478(59)	2722(54)	3564(70)	12,237(50)
missing	243(10)	384(8)	742(15)	1745(7)
Interactions with neighborhood				
Daily Mutual consultation and cooperation	359(14)	805(16)	1042(21)	4059(16)
Standing and chatting frequently	1249(50)	2679(53)	3371(67)	13,826(56)
No more than exchanging greetings	582(23)	1052(21)	1463(29)	4835(20)
None, not even greetings	54(2)	70(1)	145(3)	265(1)
missing	264(11)	451(9)	639(13)	1679(7)
Near to residence				
Locations with graffiti or garbage				
Many	99(4)	229(5)	225(4)	902(4)
Some	552(22)	1281(25)	1337(26)	6064(25)
Few	1071(43)	2389(47)	3014(60)	12,420(50)

None	334(13)	590(12)	1027(20)	3034(12)
I don't know	302(12)	363(7)	640(13)	1391(6)
missing	150(6)	205(4)	417(8)	853(3)
Parks or foot paths				
Many	284(11)	684(14)	845(17)	3502(14)
Some	1255(50)	2665(53)	3387(67)	13,495(55)
Few	532(21)	1106(22)	1394(28)	5251(21)
None	173(7)	296(6)	385(8)	1232(5)
I don't know	154(6)	163(3)	307(6)	527(2)
missing	110(4)	143(3)	342(7)	657(3)
Locations difficult for walking				
Many	225(9)	434(9)	466(9)	1435(6)
Some	865(34)	1606(32)	2055(41)	6603(27)
Few	1024(41)	2395(47)	3021(60)	13,192(53)
None	166(7)	363(7)	573(11)	2325(9)
I don't know	116(5)	132(3)	231(5)	505(2)
missing	112(4)	127(3)	314(6)	604(2)
Risky roads or crossroads for traffic accidents				
Many	337(13)	736(15)	703(14)	2927(12)
Some	1348(54)	2730(54)	3409(67)	13,297(54)
Few	562(22)	1274(25)	1854(37)	6900(28)
None	25(1)	51(1)	119(2)	342(1)
I don't know	130(5)	128(3)	266(5)	568(2)
missing	106(4)	138(3)	309(6)	630(3)
Fascinating views or buildings				
Many	138(6)	308(6)	343(7)	1418(6)
Some	756(30)	1574(31)	2066(41)	8122(33)
Few	970(39)	2185(43)	2740(54)	10,934(44)
None	263(10)	530(10)	602(12)	2365(10)
I don't know	225(9)	278(5)	476(9)	993(4)
missing	156(6)	182(4)	433(9)	832(3)
Shops selling fresh fruits and vegetables				
Many	275(11)	715(14)	832(16)	3823(16)
Some	1363(54)	2880(57)	3655(72)	14,470(59)
Few	513(20)	937(19)	1312(26)	4234(17)
None	186(7)	320(6)	420(8)	1320(5)
I don't know	69(3)	66(1)	148(3)	215(1)
missing	102(4)	139(3)	293(6)	602(2)
Dangerous places walking alone at night				
Many	329(13)	658(13)	597(12)	2372(10)
Some	1138(45)	2494(49)	2925(58)	12,319(50)
Few	533(21)	1283(25)	1941(38)	7370(30)
None	36(1)	64(1)	157(3)	357(1)
I don't know	344(14)	389(8)	680(13)	1522(6)
missing	128(5)	169(3)	360(7)	724(3)
Comfortable houses or facilities				
Many	30(1)	98(2)	136(3)	508(2)
Some	766(31)	1712(34)	2242(44)	8951(36)
Few	982(39)	2179(43)	2716(54)	10,599(43)
None	298(12)	524(10)	590(12)	2183(9)
I don't know	304(12)	388(8)	635(13)	1665(7)
missing	128(5)	156(3)	341(7)	758(3)
Someone listening to your concerns				
None	2094(83)	4469(88)	5690(113)	22,102(90)
Yes	224(9)	303(6)	424(8)	1250(5)
missing	190(8)	285(6)	546(11)	1312(5)
Someone looking after in case of ill				
None	2134(85)	4526(89)	5877(116)	22,444(91)
Yes	194(8)	276(5)	319(6)	997(4)
missing	180(7)	255(5)	464(9)	1223(5)
Attendance				
Sports group or club				
Almost everyday	19(1)	84(2)	72(1)	506(2)

Two or three times a week	85(3)	319(6)	286(6)	1866(8)
Once a week	86(3)	263(5)	219(4)	1447(6)
Once or twice a month	39(2)	150(3)	157(3)	1070(4)
A few times a year	36(1)	150(3)	152(3)	975(4)
Never	1590(63)	2920(58)	4001(79)	13,886(56)
missing	653(26)	1171(23)	1773(35)	4914(20)
Leisure activity group				
Almost everyday	17(1)	78(2)	76(2)	436(2)
Two or three times a week	115(5)	398(8)	362(7)	2071(8)
Once a week	171(7)	445(9)	489(10)	2484(10)
Once or twice a month	204(8)	571(11)	567(11)	3072(12)
A few times a year	113(5)	364(7)	346(7)	1980(8)
Never	1293(52)	2168(43)	3207(63)	10,402(42)
missing	595(24)	1033(20)	1613(32)	4219(17)

Data are given as No. (%) unless otherwise noted. Complex multimorbidity (CMM).
